# Disseminated cysticercosis with tongue involvement: a rare case report from Nepal

**DOI:** 10.1097/MS9.0000000000001292

**Published:** 2023-09-13

**Authors:** Bipin Poudel, Shubham Shrestha, Bishuddha Bhattarai, Bishal Khatri, Anusha Acharya, Bunu Maharjan, Rabindra R. Pandey, Ashim Batajoo, Kishor Khadka, Suman Thapa, Janak Koirala

**Affiliations:** a Department of Internal Medicine, Patan Academy of Health Sciences, Lalitpur; b Department of Internal Medicine, HAMS Hospital, Kathmandu; c Lalitpur Nursing Campus, Lalitpur, Nepal

**Keywords:** cysticercosis, disseminated cysticercosis, magnetic resonance imaging, neurocysticercosis, oral cavity, tongue

## Abstract

**Introduction and importance:**

Cysticercosis is a condition in which humans are infected by the larval form of the pork tapeworm *Tenia solium*. Cysticercosis in humans is common in the cerebral tissue but rare in the tongue.

**Case presentation:**

Here, the authors report a rare case of a 38-year-old male with neurocysticercosis and cysticercosis of the tongue. The patient presented with a complaint of loss of consciousness for 4–5 min. Local examination of his oral cavity revealed a swelling of ~2×2 cm on the tongue. An MRI of the brain showed various stages of neurocysticercosis involving the neuroparenchyma and tongue. For this, he was started on low-dose prednisolone of 50 mg tapered over 6 weeks and levetiracetam of 500 mg BD continued for his seizure episodes. He is responding well with the medications and is planned to start antiparasitic agent only after the perilesional edema decreases.

**Clinical discussion:**

Cysticercosis may involve the central nervous system, muscle, heart, lungs, peritoneum, eye, and subcutaneous tissue. Oral cavity and perioral involvement by cysticercous larva is rare in humans. Radiologic imaging, serology, and tissue biopsy can be used to confirm a diagnosis of cysticercosis. The most common locations for oral cysticercosis are the tongue, buccal mucosa, lower lip, and upper lip.

Only 102 cases of oral cysticercosis have been reported based on a PubMed English-language literature search.

**Conclusion:**

Oral cysticercosis is a rare event, and it represents a difficulty in clinical diagnosis. But a patient with a mass in the tongue should be considered as a possible case of cysticercosis especially in endemic regions like Nepal.

## Introduction

Highlights

- Cysticercosis in humans is common in the cerebral tissue but rare in the tongue.
- The most common locations for oral cysticercosis are the tongue, buccal mucosa, lower lip, and upper lip.
- A Patient with a mass in the tongue should be considered as a possible case of cysticercosis.

Cysticercosis is a condition in which humans are infected by the larval form of the pork tapeworm *Tenia solium* and results from the ingestion of tapeworm eggs through contaminated food and water or dirty hands, humans acting as the intermediate host. The adult *T. solium*, or pork tapeworm, lives in the small intestine of man, its definitive host. Humans act as both definitive and intermediate hosts of *T. solium* ^1^.

Cysticercosis in humans is common in the cerebral tissue, subcutaneous tissue, muscle, and the eye^2^. The oral cavity is a rare site of involvement by cysticercosis, even in an endemic area^3^. In addition, cysticercosis presenting as a nodule or mass in the tongue is even more rare^4^. Only 102 cases of oral cysticercosis have been reported in the worldwide based on a PubMed English-language literature search. We present a rare case of neurocysticercosis along with cysticercosis of the tongue. This case has been reported following the Surgical CAse REport (SCARE) criteria^5^.

## Case report

**Figure 1:**
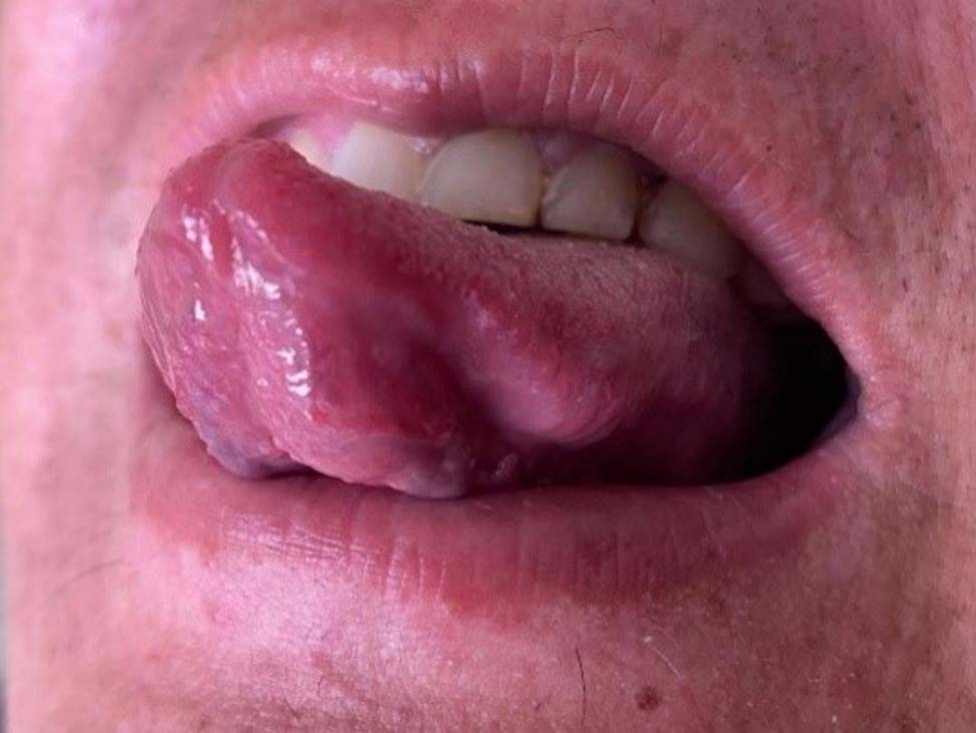
Swelling on ventral aspect of tongue.

A 38-year-old male initially presented to the Medicine Outpatient Department in a tertiary care center of Nepal with a complaint of loss of consciousness (LOC) for 4–5 min. Loss of consciousness was associated with clenching of teeth, up rolling of eyes, vacant stare, and frothing from the mouth, which was suggestive of a seizure. He had experienced similar episodes of symptoms a year back. In addition, he also had painless swelling over the ventral aspect of the anterior 1/3rd of the tongue as shown in Figure 1. He denied bowel and bladder incontinence, or change in his behavior. He is a diagnosed case of hypertension under amlodipine and losartan. Regarding dietary habits, he is a nonvegetarian who has been consuming pork meat for 10 years as well as he is an active smoker and drinker.

There was no history of hoarseness of voice, dysphagia/odynophagia, nonhealing oral ulcers, or weight loss. There was no history of headaches, localized sensory/motor deficits, parasthesias, and personality changes.

**Figure 2:**
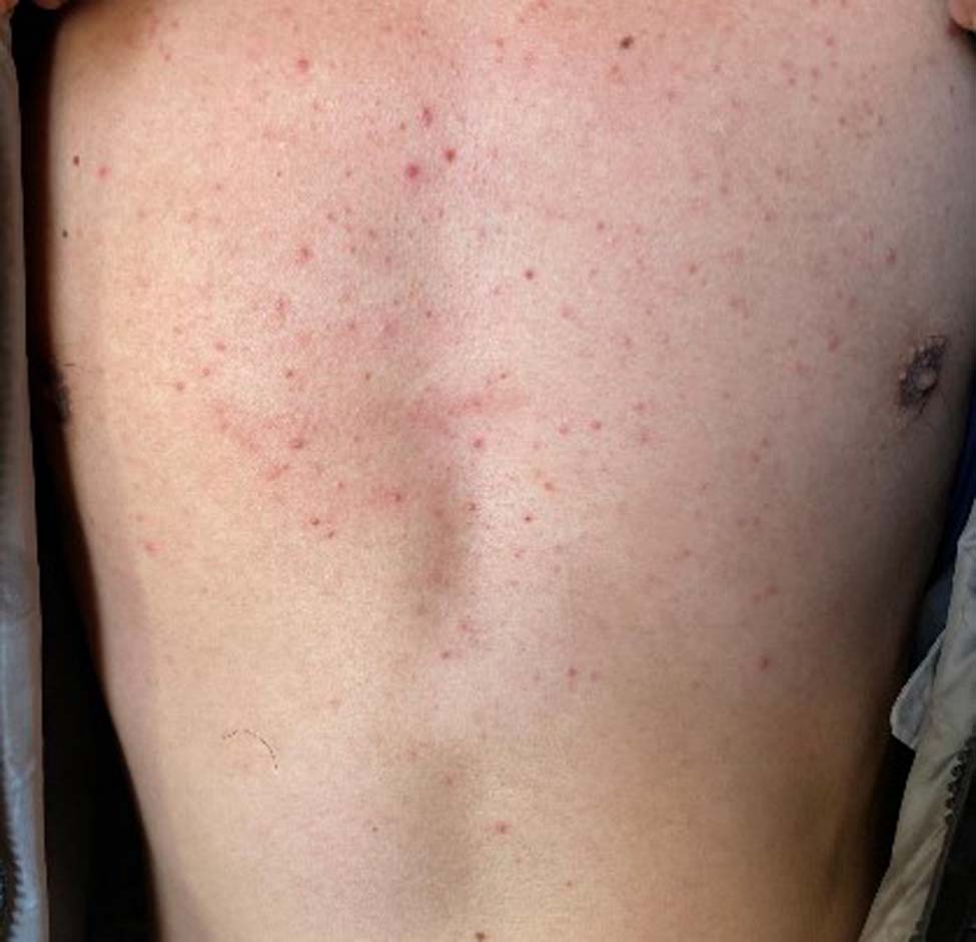
Multiple erythematous lesions in chest region.

Local examination of his oral cavity revealed swelling of ~2×2 cm which was firm, mobile, nontender with a diffuse margin, and normal mucosa with unrestricted tongue movement. On chest examination, multiple nontender erythematous lesions were present as shown in Figure 2 with normal other systemic examinations.

**Figure 3:**
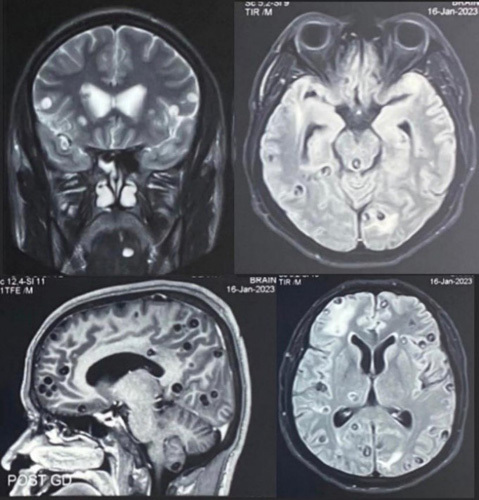
MRI brain showing innumerable cystic lesions throughout cerebral hemisphere and within tongue muscles giving starry sky appearance.

**Figure 4:**
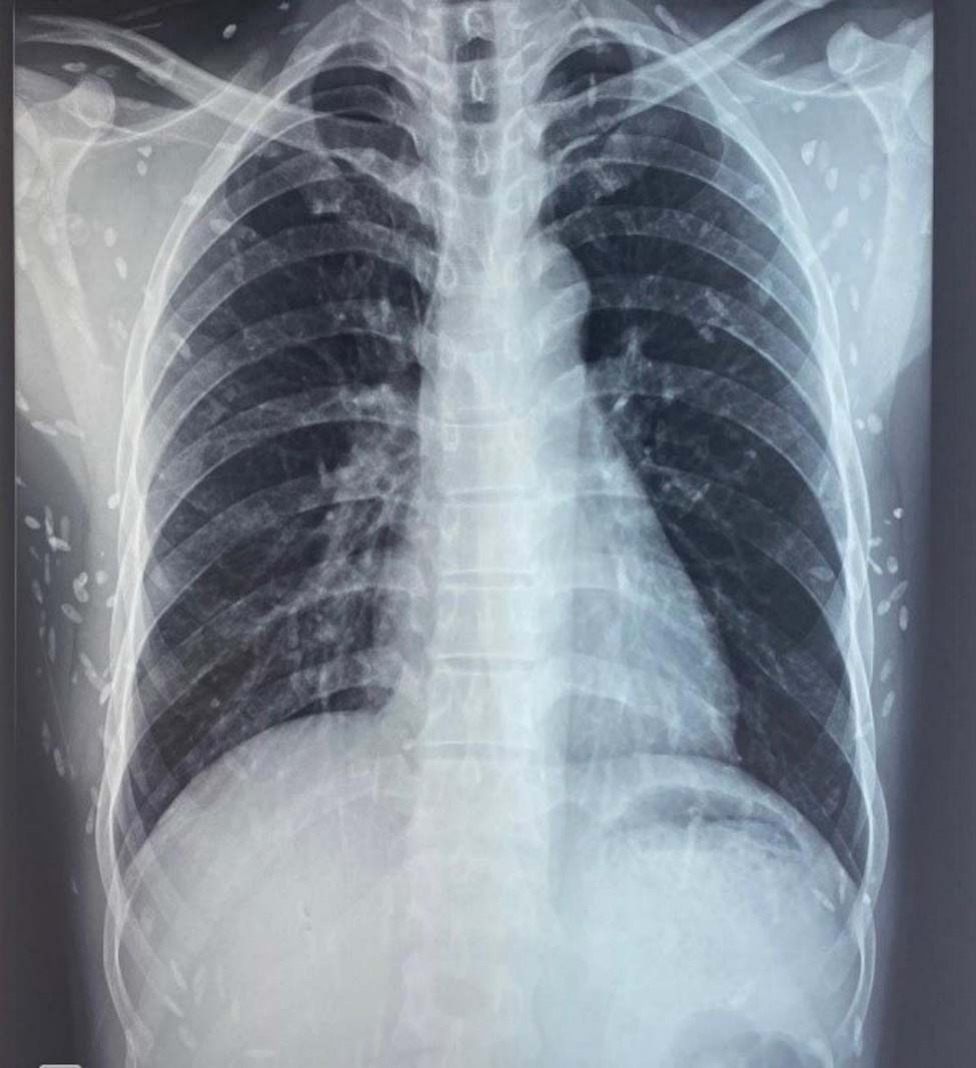
CXR showing multiple grain like white lesions.

His blood reports were within the normal range. A chest radiograph showed multiple grain like lesion present all over the chest muscles as shown in Figure 3. On doing further work-up for his seizure, MRI of the brain showed various stages of neurocysticercosis involving neuroparenchyma and tongue as shown in Figure 4. T1 hypointense and T2 hyperintense foci were seen in the neuroparenchyma with a central dot confirming neurocysticercosis. Some of the cysts showed edema in the surrounding parenchyma and some did not. Finally, this case was diagnosed as a mixed infection of neurocysticercosis and cysticercosis of the tongue. For this, he was started on low-dose prednisolone of 50 mg tapered over 6 weeks and levetiracetam of 500 mg BD continued for his seizure episodes. On follow-up, he was found to be compliant and responding well with the medications. The patient had disseminated neurocysticercosis and pre-existing edema and cysts throughout the brain parenchyma in different stages of maturation. So, patient is planned to start antiparasitic agent only after the perilesional edema decreases.

## Discussion

Cysticercosis is caused by the larval form of *T. solium*, a hermaphroditic parasite^6^. Humans act as both intermediate and definitive hosts, while pigs are intermediate hosts. Eggs ingested by pigs from human feces-contaminated soil then release embryos that penetrate the intestinal wall and develop into larval form (cysticercosis). Humans acquire these larvae by consuming uncooked pork, completing *T. solium’s* life cycle in the small intestine^7^. These infected humans can have taeniasis for long periods and contaminate the environment continuously^8^.

Cysticercosis develops when humans ingest tapeworm eggs instead of pigs; humans acting as an intermediate host. This usually happens by consumption of faecally contaminated food and water or by faeco oral contamination as well as by reflux of eggs into the stomach^9^. The eggs develop into oncospheres that penetrate the intestine wall and – via lymphatic or vascular circulation – reach a destination, where larvae develop and become the cysticerci or ʻa fluid-filled cystʼ. Once a person becomes the host of cysticercus cellulosae, cysticercosis can develop in various organs and tissues^2^.

The most serious involvement is that of the central nervous system called as neurocysticercosis, followed by ocular involvement, usually the only ones which are symptomatic^8^. Neural cysticercosis has nonspecific signs and symptoms; however, acute symptomatic seizures is the most common presentation. Our case too had presentation of seizure for which levetiracetam of 500 mg BD was prescribed.

Extraneural cysticercosis, which is also very common, infects muscles, the heart, lung, peritoneum, eye, and subcutaneous tissue^10^. Oral cavity and perioral involvement by cysticercous larva is uncommon in humans^11^. According to a series published by Singh *et al.* ^6^ in 2018 a total of 89 cases of oral cysticercosis (excluding masseter and parotid) had been reported in PubMed English literature, out of which 50.6% were in the tongue. Since then, 13 more case has been reported, excluding the present case making it total of 102 cases reported till date. The most common locations for oral cysticercosis are the tongue, buccal mucosa, lower lip, and upper lip^12^.

While tongue muscle involvement in cysticercosis is common in swine, it is rare in humans. The reason for this is unclear, but some suggest that the high muscular activity and metabolic rate in humans may hinder the cysticercus’ lodging and development in this location^13^. Lingual cysticercosis can be mistaken for other lesions like fibromas, lipomas, or salivary gland tumors^14^.

Radiologic imaging, serology, and tissue biopsy can be used to confirm a diagnosis of cysticercosis. Imaging techniques, in particular computed tomography and MRI, are of great value to diagnose cerebral cysticercosis. Histopathological examination makes up a diagnosis of cysticercosis by the detection of a cystic space containing the cysticercus cellulosae^7^. In most cases diagnostic certainty is not possible; instead a clinical diagnosis is made on the basis of a combination of clinical presentation, radiographic studies, serologic tests, and exposure history^15^ In contrast to our case, serology and immunology tests for cysticercosis are not available in the center, and when done in other private labs are expensive for the average Nepali (for which the patient has to pay out of pocket). Given the good utility provided by the MRI; the treating team decided further testing would add financial burden to the patient without necessarily providing additional information or influencing the treatment. So the tests were not done.

The treatment of oral cysticercosis is surgical excision. Praziquantel and albendazole are used to treat cysticercosis, especially in patient with disseminated cysticercosis or where surgical excision is risky or not possible, such as in neurocysticercosis^16^. However, cysticidal drugs may be associated with severe reactions such as local tissue swelling and generalized anaphylactic reaction due to massive antigen release. The use of corticosteroids with or before their administration can help in decreasing the incidence of these complications^17^ In contrast to this, in our case, the patient was started on low-dose prednisolone of 50 mg tapered over 6 weeks and levetiracetam of 500 mg BD continued for his seizure episodes. He is responding well with the medications and is planned to start antiparasitic agent only after the perilesional edema decreases.

The risk of *T. solium* cysticercosis is linked to the consumption of contaminated food and the practice of coprophagy. Therefore, it is important to inspect pork and ensure proper hygiene and sanitation measures are in place, including the appropriate disposal of human feces^18^. Health education plays a crucial role in raising awareness about preventing tapeworm infection through adequate pork cooking. Additionally, promoting good personal hygiene and proper fecal disposal can significantly reduce the occurrence of cysticercosis^19^.

## Conclusion

Oral cysticercosis is a rare event, and it represents a difficulty in clinical diagnosis. But a patient with a mass in the tongue should be considered as a possible case of cysticercosis especially in endemic regions like Nepal. This case is about a mixed case of neurocysicercosis and cysticercosis of a tongue managed conservatively. We expect this case could raise the attentions to the control of *T. solium* infection and subsequent cysticercosis.

## Ethical approval

None. Since this report involves no experiments, the ethical approval is waived.

## Consent

Written informed consent was obtained from the patient for publication of case report. A copy of the written consent is available for review by the Editor-in-Chief of this journal on request.

## Sources of funding

None.

## Author contribution

All authors have contributed to the writing, editing, and preparation of the manuscript and have reviewed it before submission.

## Conflicts of interest disclosure

The authors declare that they have no financial conflicts of interest with regard to the content of this report.

## Research registration unique identifying number (UIN)

1. Name of the registry: not applicable.

2. Unique identifying number or registration ID: not applicable.

3.Hyperlink to your specific registration (must be publicly accessible and will be checked): not applicable.

## Guarantor

Bipin Poudel.

## Data availability statement

None.

## Provenance and peer review

Not commissioned, externally peer-reviewed.
